# Cross-sectional associations between cognition and mobility in Parkinson’s disease

**DOI:** 10.1590/1980-57642021dn15-010011

**Published:** 2021

**Authors:** Nariana Mattos Figueiredo Sousa, Roberta Correa Macedo, Sonia Maria Dozzi Brucki

**Affiliations:** 1 Neurorehabilitation Program, Rede SARAH de Hospitais de Reabilitação – Salvador, BA, Brazil.; 2 Department of Neurology, Faculdade de Medicina, Universidade de São Paulo – São Paulo, SP, Brazil.

**Keywords:** Parkinson’s disease, gait, cognitive impairment, balance, executive functions, doença de Parkinson, marcha, comprometimento cognitivo, equilíbrio, funções executivas

## Abstract

Cross-sectional studies show an association of decline in mental flexibility and inhibitory control with reduced gait speed and falls, as well as divided attention deficit and difficulty in initiating gait.

**Objective:**

To investigate the relationships between cognitive function and gait performance in patients with Parkinson’s disease (PD) who participated in a hospital neurorehabilitation program.

**Methods:**

A total of 107 patients (79 males, 28 females; mean age 61.00±8.2 years; mean schooling 11.7±4.1 years) with idiopathic PD (mean disease duration 5.5±4.1 years) were recruited for this study. Among them, 78.50% were in stages I and II of the Hoehn & Yahr Scale. Cognitive functions were evaluated through the Digit Span test, Trail Making Test, and Addenbrooke’s Cognitive Examination III. Motor function was assessed with the 10-Meter Walk Test, the short version of the Balance Evaluation Systems Test (Mini-BESTest), and the Timed Up and Go Test.

**Results:**

Balance skills were significantly correlated with global cognition and specific domains, including divided attention, verbal fluency, and visuospatial function. Functional mobility showed a significant association with all cognitive tests, except for the number of errors on TMT-A. Gait speed presented a significant correlation with global cognition scores, memory, and attention, including divided attention.

**Conclusions:**

These findings might help early identification of cognitive deficits or motor dysfunctions in PD patients who may benefit from rehabilitation strategies, as well as facilitate fall risk assessments and strategies to prevent falls. Future prospective studies are needed to investigate the effects of cognitive training on motor performance, since the difficulty in motor rehabilitation may be more related to cognitive loss than to motor damage.

## RESUMO.

Estudos transversais mostram associação entre declínio da flexibilidade mental e controle inibitório com redução da velocidade de marcha e quedas, assim como déficit de atenção dividida e dificuldade para iniciar a marcha.

**Objetivo:**

Investigar as relações entre a função cognitiva e o desempenho da marcha em pacientes com Doença de Parkinson (DP) que participaram de um programa de reabilitação hospitalar.

**Métodos:**

Um total de 107 pacientes (79 homens, 28 mulheres; idade média de 61,00±8,2 anos, média de escolaridade 11,7±4,1) apresentando DP idiopática (duração média da doença: 5,5±4,1 anos) foram recrutados para o estudo. Desses, 78,50% estavam nos estágios I e II da Hoehn e Yahr. As funções cognitivas foram avaliadas por meio do teste de Dígitos Spam, Teste de Trilhas e Exame Cognitivo de Addenbrooke (terceira versão). A função motora foi examinada por meio do teste de caminhada de 10 metros, Mini BESTest e teste *Timed Up and Go*.

**Resultados:**

As análises de correlação mostraram que as habilidades de equilíbrio estavam significativamente correlacionadas com a cognição global e com domínios específicos, incluindo atenção dividida, fluência verbal e função visuoespacial. Além disso, a mobilidade funcional apresentou correlação significativa com todos os testes cognitivos, exceto TMT-A (erro). A velocidade da marcha mostrou correlação significativa com escores globais de cognição, memória e atenção, incluindo atenção dividida.

**Conclusões:**

Esses achados podem ajudar na identificação precoce de déficits cognitivos ou disfunções motoras em pacientes com DP que podem se beneficiar de estratégias de reabilitação, facilitar avaliações de risco de queda e estratégias de prevenção de queda. Estudos prospectivos futuros são necessários para investigar os efeitos do treino cognitivo no desempenho motor, uma vez que a dificuldade na reabilitação motora pode estar mais relacionada à perda cognitiva do que aos prejuízos motores.

## INTRODUCTION

Parkinson’s disease (PD) is the second most common neurodegenerative disease, which has cognitive impairment as a prevalent and debilitating non-motor symptom. Non-motor symptoms, such as disturbances of the autonomic nervous system, sleep disorders, depression, and cognitive and neuropsychiatric disorders, can precede motor symptoms or appear throughout the disease, impacting the functional independence of the patient.^1^ ^,^ ^2^

Cross-sectional and longitudinal studies^3^ ^–^ ^5^ have reported that gait changes may be associated with cognitive impairment, particularly executive functions. Additional investigations have also revealed that slow gait speed predicted cognitive impairment and dementia.^6^ However, some studies have suggested that cognitive impairment preceded gait changes.^7^ ^,^ ^8^

Studies that correlated the performance on the Montreal Cognitive Assessment (MoCA) with motor tests have found a significant association with dexterity and mobility evaluated through the Purdue Pegboard Test and the Timed Up and Go Test (TUG). Furthermore, although no correlation was found between the tremor dominant subtype and cognitive impairment, the postural instability/gait difficulty (PIGD) subtype showed an association with lower performance on cognitive tests.^9^ ^–^ ^12^

Associations between gait and cognition in PD indicate an influence of attention and executive functions on the gait pace and variability.^13^ Another study^14^ argues whether the impact of cognition on gait performance can be so specific that data collected by wearable devices can contribute to differentiating dementia subtypes.

Consistent findings have shown the limited ability of this population to cope with complex tasks that require cognitive demands, namely, recognizing and avoiding obstacles, dividing attention while walking.^15^ The ability to plan and monitor gait adequately while dealing with cognitive overload, as well as when performing dual-tasking (DT), is impaired in this population.^16^ ^–^ ^18^ Carrying out two tasks simultaneously is difficult for these individuals.^19^ Executive function deficit, mainly inhibitory control and mental flexibility, has been associated with gait impairment and freezing of gait (FOG).^11^ ^,^ ^20^ ^,^ ^21^ Another study revealed that gait and balance are related to specific cognitive skills, suggesting similar cerebral cortical circuitry for mobility and cognitive function.^13^

Cognitive control is essential for gait; cognitive issues are risk factors for poor gait performance, especially falls, consequently limiting the community participation in daily activities. Balance skills were significantly correlated with the ability to divide attention and the visuospatial ability in a recent study conducted by these authors.^22^ In addition, several investigations have reported that gait performance, risk of falling, freezing, and PD stage were strongly and significantly associated with DT.^23^ ^,^ ^24^

Thus, the present study aimed to examine the association between cognitive ability, including global and specific cognitive functions (i.e., executive function, visuospatial ability, attention, language, and memory), and gait performance in PD patients. In this study, the characteristics of the sample assessed, that is, a homogeneous group as to cognitive profile (patients with mild cognitive impairment), as well as the sample size, contributed to the accuracy of the analyses.

## METHODS

### Participants

This is a cross-sectional study of 107 patients diagnosed with PD, according to the UK Brain Bank criteria.^25^ The subjects were part of an outpatient neurorehabilitation program at the SARAH Network of Rehabilitation Hospitals.

The inclusion criteria were: idiopathic PD patients aged over 50 years, more than 4 years of schooling, no psychiatric disorders before the PD diagnosis, as well as no history of substance use and abuse, absence of behavioral, motor, and/or sensory changes that may interfere with the performance of the cognitive tests (patients evaluated in the ON state, with no motor fluctuation during the assessment). Patients with moderate or severe depressive symptoms (Beck Depression Inventory — BDI≥20),^26^ Hoehn and Yahr Scale (H&Y) stage IV, and dementia, according to the Movement Disorder Society guidelines,^27^ were excluded.

All participants signed the informed consent form. The local ethics committee approved this study.

### Cognitive evaluation

The patients were evaluated on their global and specific cognitive functions by a neuropsychologist.

- Digit Span Test (forward and backward) (Wechsler Memory Scale-revised — WMS-R):^28^ evaluates the immediate memory capacity and the ability to manipulate information.
- Trail Making Test (TMT) — A and B:^29^ assesses visuomotor speed, selective attention, and mental flexibility.
- Addenbrooke’s Cognitive Examination-third version (ACE-III):^30^ focuses on the global score and subscores (attention/orientation, memory, verbal fluency, visuospatial ability, and language). The maximum score is 100.

### Motor evaluation

- 10-Meter Walk Test (10MWT): measures walking speed in meters per second while the subject walks 10 meters. The participants were asked to walk at a comfortable pace.^31^
- Short version of the Balance Evaluation System Test (Mini-BESTest): assesses balance through 14 tasks (anticipatory postural adjustments, reactive postural control, sensory orientation, gait, and dynamic balance). Each task is graded according to the performance on a 3-level ordinal scale (0–2), and the maximum score is 28 points in case of normal performance.^32^
- Timed Up and Go Test (TUG test): evaluates mobility and balance, calculating the time that a person takes to rise from an armchair, walk three meters, turn around, walk back, and sit down in the chair. In the cognitive TUG, the individual performs the same motor task plus a second motor task or cognitive task concomitantly. In this study, subjects were asked to complete the TUG associated with mathematical tasks requested by the examiner.^33^ This test was scored following the same Mini-BESTest scale (0–2), according to the impact of the cognitive task on motor performance.

### Data analysis

*Statistical Package for the Social Sciences* (SPSS) software, version 22.0, was used for data analysis. Descriptive analysis of the study participants was expressed as mean and standard deviation (SD). Pearson’s correlation was applied to assess the association between gait parameters (speed, balance, and functional mobility) and cognitive scores (total and per domains). Some variables (such as age and disease duration and severity) could have skewed these results. A multivariate linear regression analysis was performed to minimize these effects. The probability level was set at 0.05 to determine significance.

## RESULTS

### Demographic and clinical characteristics

The study included 107 participants (79 male and 28 female). Among them, 84 had mild impairment (stages I–II of H&Y), that is, no patients had less severe disease. PD patients had an average disease duration of 5.5±4.1 years. Table 1 presents their demographic and clinical characteristics.

**Table 1:** Demographic and clinical characteristics of participants.

n=107		Mean±SD/n (%)
Age (years)		61.00±8.2
Gender (male/female)		79 (73.83)/28 (21.4)
Schooling (years)		11.7±4.1
Disease duration (years)		5.5±4.1
Hoehn & Yahr Scale		
	I–II	84 (78.50)
	III–IV	23 (21.49)
BDI		6.3±4.8

SD: standard deviation; BDI: Beck Depression Inventory.

### Cognitive and motor performance

Table 2 shows the descriptive data from cognitive and motor performance.

**Table 2:** Descriptive statistics of cognitive and motor tests.

	n=107	Mean±SD/n (%)	Minimum	Maximum
ACE-III/Total score		85.07±11.30	54	99
	Attention/Orientation	16.36±1.78	11	18
	Memory	19.64±4.73	4	26
	Fluency	9.97±2.70	2	14
	Language	25.00±2.17	11	26
	Visuospatial	14.09±2.60	3	16
Digit Span (forward)		5.52±4.47	3	7
Digit Span (backward)		3.62±1.02	2	5
TMT-A (s)		68.93±29.79	0	189
TMT-A (errors)		1.57±1.19	0	4
TMT-B (s)		197.40±108.68	1	654
TMT-B (errors)		1.19±1.57	0	6
TMT (A–B)		−128.5±95.0	−1	−573
10MWT		294±23.98	11	200
Mini-BESTest		22.39±4.23	6	28
TUG 0 or 1		74 (69.15)	−	−
TUG 2		33 (30.84)	−	−

SD: standard deviation; ACE-III: Addenbrooke’s Cognitive Examination; TMT: Trail Making Test; 10MWT: 10-Meter Walk Test; Mini-BESTest: Mini-Balance Evaluation Systems Test; TUG: Timed Up and Go Test.

### Associations between gait and cognition

Significant associations were found between TUG scores and ACE-III total and domain scores, in addition to other neuropsychological tests, except for TMT-A errors (r=−0.0686, p=0.4827). The Mini-BESTest also demonstrated a significant correlation with cognitive measures. Correlations were identified between 10MWT and ACE-III total and memory scores, as well completion time for TMT-A and B (Table 3).

**Table 3:** Correlations between cognitive and motor variables.

Variables		Mini-BESTest	TUG	10MWT (cm/s)
Total score (ACE-III)		r=0.2231	0.4876	0.2152
		p=0.0209*	0.0000***	0.0260*
	Attention/Orientation	r=0.1126	0.4117	0.0405
		p=0.2481	0.0000***	0.6791
	Memory	r=0.1658	0.4094	0.2857
		p=0.0878	0.0000***	0.0029**
	Fluency	r=0.1910	0.3906	0.1673
		p=0.0488*	0.0000**	0.0849
	Language	r=0.0899	0.2645	0.1791
		p=0.3573	0.0059**	0.0649
	Visuospatial	r=0.2642	0.3874	0.1835
		p=0.0060**	0.0000***	0.0585
TMT-A (s)		r=-0.1981	−0.4420	−0.2044
		p=0.0408*	0.0000***	0.0347*
TMT-A (errors)		r=0.0132	−0.0686	−0.1297
		p=0.8928	0.4827	0.1832
TMT-B (s)		r=-0.3024	−0.4701	−0.2291
		p=0.0015**	0.0000***	0.0176*
TMT-B (errors)		r=-0.2315	−0.3366	0.1117
		p=0.0170*	0.0004***	0.2542
TMT (B-A)		r=0.2633	r=0.3689	r=0.1815
		p=0.0061**	p=0.0001***	p=0.0614
Digit Span (forward)		r=0.1912	0.3811	0.0697
		p=0.0486*	0.0001***	0.4758
Digit Span (backward)		r=0.1354	0.3757	0.1660
		p=0.1645	0.0001***	0.0874
BDI		r=-0.1007	−0.1133	−0.0476
		p=0.3021	0.2454	0.6260
H&Y		r=-0.4615	−0.3635	−0.1766
		p=0.0000***	0.0001***	0.0689

ACE-III: Addenbrooke’s Cognitive Examination; TMT: Trail Making Test; H&Y: Hoehn & Yahr Scale; BDI: Beck Depression Inventory; Mini-BESTest: Mini-Balance Evaluation Systems Test; TUG: Timed Up and Go Test; 10MWT: 10-Meter Walk Test; r: Pearson’s correlation coefficient.

* significant at p<0.05;

** significant at p<0.01;

*** significant at p<0.001.

Table 4 reveals that the ACE-III total score was associated with cognitive TUG (β=0.01918, p=0.009), disease severity (evaluated by H&Y), Mini-BESTest (β=−2.74613, p=0.000), and TUG (β=−0.25986, p=0.004).

**Table 4:** Multiple linear regression analysis.

Dependent variables		ACE-Total	Digit Span (forward)	Digit Span (backward)	TMT-A (s)	TMT-B (s)	Disease duration	H&Y
TUG								
	β	0.01918	0.0092051	0.0096998	−0.002354	−0.000338	0.001427	−0.25986
	p	0.009**	0.463	0.896	0.349	0.562	0.915	0.004**
Mini-BESTest								
	β	−0.06708	0.14169	−0.403646	0.007995	−0.034887	−0.06364	−2.74613
	p	0.196	0.115	0.445	0.656	0.403	0.000***	0.000***
10MWT								
	β	−0.46584	0.721661	1.53593	−0.01004	0.05360	−0.28694	−5.19196
	p	0.147	0.193	0.638	0.928	0.835	0.625	0.183

ACE: Addenbrooke’s Cognitive Examination; TMT: Trail Making Test; H&Y: Hoehn & Yahr Scale; TUG: Timed Up and Go Test; Mini-BESTest: Mini-Balance Evaluation Systems Test; 10MWT: 10-Meter Walk Test.

* significant at p<0.05;

** significant at p<0.01;

*** significant at p<0.001.

Secondary analyses were carried out, with the formation of subgroups of aspects such as age and disease duration and severity, since these factors can interfere with the association between gait and cognition variables. The results were the same when correlated with the functional mobility test (TUG).

Table 5 indicates a significant correlation between the TUG test and several cognitive tasks, regardless of the sample stratification by the median. Regarding other motor tests (Mini-BESTest and 10MWT), the association was present in younger individuals and those whose disease diagnosis was more recent (shorter disease duration).

**Table 5:** Pearson’s correlation coefficient. Subgroup: Timed Up and Go Test.

	Age (y)	Age (y)	Disease (y)	Disease (y)	H&Y	H&Y
	<60	≥60	<5	≥5	I–II	III
ACE (total score)	r=0.4189	0.5544	0.5276	0.4504	0.4690	0.4472
	p=0.0000***	0.0000***	0.0000**	0.0010**	0.000***	0.0250*
Attention/Orientation	r=0.2506	0.5740	0.4319	0.4362	0.3845	0.2620
	p=0.0732	0.0000***	0.0008***	0.0015**	0.0004***	0.2058
Memory	r=0.4007	0.4242	0.3979	0.4608	0.3933	0.4255
	p=0.0032**	0.0012**	0.0022**	0.0008***	0.003**	0.0340*
Fluency	r=0.3467	0.4133	0.5049	0.2993	0.3810	0.3805
	p=0.0118*	0.0017**	0.0001***	0.034*	0.0004***	0.0606
Language	r=0.2110	0.3184	0.2493	0.2330	0.2697	0.1709
	p=0.1333	0.0178*	0.0614	0.1035	0.0143*	0.4140
Visuospatial	r=0.3232	0.4164	0.5003	0.2019	0.3533	0.4920
	p=0.0194*	0.0016**	0.0001***	0.1596	0.0111*	0.0125*
TMT-A (s)	r=-0.4446	−0.4665	−0.5077	−0.3249	−0.3800	0.4379
	p=0.0010**	0.0003***	0.0001***	0.0213*	0.0004***	0.0286*
TMT-A (errors)	r=-0.0537	−0.0530	−0.1221	−0.0241	−0.0536	0.2705
	p=0.7052	0.7006	0.3657	0.8682	0.6324	0.1909
TMT-B (s)	r=-0.4506	−0.4758	−0.5150	−0.3576	−0.4340	0.4239
	p=0.0008***	0.0002***	0.000***	0.0108*	0.0000***	0.0347*
TMT-B (errors)	r=-0.3098	−0.3518	−0.4989	−0.0754	−0.3125	0.3481
	p=0.0254*	0.0091**	0.0001***	0.6026	0.0045**	0.0882
TMT (A-B)	r=0.3561	0.3573	0.4178	0.2774	0.3769	0.2052
	p=0.0096**	0.0074**	0.0012**	0.0511	0.0005***	0.3251
Digit Span (forward)	r=0.4120	0.3811	0.3546	0.4365	0.3365	0.4129
	p=0.0024**	0.0041**	0.0068**	0.0015**	0.0020**	0.0402*
Digit Span (backward)	r=0.3754	0.3736	0.4132	0.2976	0.3031	0.4114
	p=0.0061**	0.0050**	0.0014**	0.0358*	0.0056*	0.0411*

ACE-III: Addenbrooke’s Cognitive Examination; TMT: Trail Making Test; H&Y: Hoehn & Yahr Scale;

* significant at p<0.05;

** significant at p<0.01;

*** significant at p<0.001.

## DISCUSSION

The present study aimed to determine whether cognitive functions were associated with gait performance, mobility, and balance in idiopathic PD. The findings revealed that motor parameters were significantly related to cognitive skills, especially aspects connected to executive functions and global cognition, evidencing that balance ability and functional mobility were significantly correlated with the ability to divide attention between tasks performed at the same time, as found in similar research.^17^ ^,^ ^18^ This interaction has been identified in cognitive tests that assess mental flexibility (TMT-B and TMT B-A), attention (Digit Span forward), working memory (Digit Span backward), and functional mobility (cognitive TUG). The correlation between the simultaneous performance of a functional mobility activity and a mental task increased with cognitive dysfunction.

Cognitive decline, especially in executive functions, has been associated with gait disorders and risk of falling.^16^ ^,^ ^20^ The association between executive functions and bradykinesia has been reported in individuals with PD,^3^ ^,^ ^18^ as well as the correlation between the ability to divide attention between two tasks and balance.^30^

A study that researched the relationship between cognition, emotion, and motor function found that those who presented higher freezing rates had worse performance on the Wisconsin Card Sorting Test, TMT-A, and Rey Auditory Verbal Learning Test, indicating an association with tests of executive functions and attention/processing speed.^20^

A previous investigation showed a correlation between a functional mobility motor test (TUG) and cognition. However, no correlation between cognitive variables and gait speed test was found. After increasing the sample, the 10MWT also demonstrated a significant association with some cognitive variables, suggesting that impaired cognitive function might be related to slower gait speed. TUG continued to be the motor test with the best correlation, followed by the Mini-BESTest.^22^

As real-world activities usually involve the combination of motor and cognitive tasks, the performance of the TUG test with a cognitive task has been chosen as a more sensitive outcome measure to predict the risk of falling. Thus, the cognitive TUG test has been recommended as an essential outcome measure to evaluate mobility and risk of falling.^34^ Moreover, a retrospective cohort study of individuals with PD investigated the impact of adding a task (manual or cognitive) to the TUG test and concluded that it increases the responsiveness of the test in detecting the risk of falling.^35^ Therefore, the role played by attention skills in safety, effectiveness, and independence during the performance of motor tasks is evident, especially when multiple stimuli are competing with this activity, which is usual when it comes to community participation.

The present results are consistent with a previous cross-sectional study that also reports an association between changes in gait and cognitive decline.^14^ ^,^ ^20^

Finally, this research offers some ideas for the assessment, referral, and holistic treatment of PD patients, since the individual who seeks the medical or physical therapy service with gait and mobility deficits may be eligible for a cognitive assessment and personalized rehabilitation program that covers not only the motor aspects. This interdisciplinary and biopsychosocial approach is also acknowledged in two recent meta-analyses.^36^ ^,^ ^37^

This study has several strengths: neuropsychological evaluation with standardized cognitive measures, patients with similar cognitive characteristics (mild cognitive impairment), motor assessment through a quantitative tool. It also has some limitations, such as evaluating only gait speed, without other measures like cadence and single or double support time.

In conclusion, the present findings suggest the critical role played by divided attention, visuospatial ability, and mental flexibility in the gait of PD patients. Thus, individuals with impaired mobility should not only be evaluated by a neuropsychologist but also have a personalized rehabilitation plan involving motor and cognitive training, when possible.
